# Large intradiploic arachnoid cyst of the skull in child—a case report and new terminology proposition

**DOI:** 10.1007/s00381-023-06255-x

**Published:** 2023-12-21

**Authors:** Adrian Drożdż, Tomasz Wojciechowski, Bogdan Ciszek, Zygmunt Stopa

**Affiliations:** 1 Department of Descriptive and Clinical Anatomy, Center for Biostructure Research, Medical University of Warsaw, 5 Chałubińskiego St, 02004 Warsaw, Poland; 2 Department of Pediatric Neurosurgery, Bogdanowicz Memorial Hospital for Children, 4/24 Niekłańska St, 03924 Warsaw, Poland; 3 Department of Maxillofacial Surgery, Medical University of Warsaw, 4 Lindleya St, 02005 Warsaw, Poland; 4 Department of Neurosurgery, Bródno Masovian Hospital, 8 Kondratowicza St, 03242 Warsaw, Poland

**Keywords:** Arachnoid membrane, CSF collection, Growing skull fracture, Intracranial cyst

## Abstract

We present a rare finding of the arachnoid matter invaginating into the base of middle cranial fossa and creating an abnormal space. Presented entity was incidentally found in head CT scan of 12-year-old male. Based on the radiological characteristics in CT scans and MR images, the diagnosis of intradiploic arachnoid cyst (AC) was suggested. After surgical intervention and histopathological analysis of the specimen, the diagnosis was confirmed. We assume this is the first description of large intrasphenoid AC without any traumatic or iatrogenic cause. The literature provides many different terms for the phenomenon. We are proposing the term *intradiploic arachnoid diverticulum* as the more accurate for capturing the essence of the phenomenon. It provides clear differentiation of the entities from classical arachnoid cysts since they are of different anatomical localization (intradural vs. extradural) and etiopathogenesis. Management with arachnoid diverticulum is not yet established, but observation with serial imaging studies should be recommended as primary management in case of asymptomatic cyst. When cyst is symptomatic, surgical treatment may be required.

## Introduction

Arachnoid cysts (ACs) are benign collections of fluid which develop inside the arachnoid mater of the cranium or vertebral column [1]. ACs represent approximately 1% of all intracranial lesions, and they are most commonly found in the middle cranial fossa in the area of Sylvian fissure [2]. When advanced radiologic tools such as computed tomography (CT) or magnetic resonance imaging (MRI) became widespread, the diagnosis of ACs started to increase [3]. There are a few possible causes of the development of ACs but no definite etiology has yet been found. While most of them are intradural, the intradiploic localization is related to the discontinuity of the dura and breaching of the arachnoid membrane [4]. Indications for surgical treatment seem to be obvious in the case of symptomatic patients, while the management of asymptomatic arachnoid cysts is still debatable. Intracystic shunt and surgical or endoscopic fenestration are most widely considered as proper interventional treatment, but intradiploic localization qualifies finding as a specific subtype and, in most cases, limits possible treatment to open surgery only.

## Clinical presentation

**Fig. 1:**
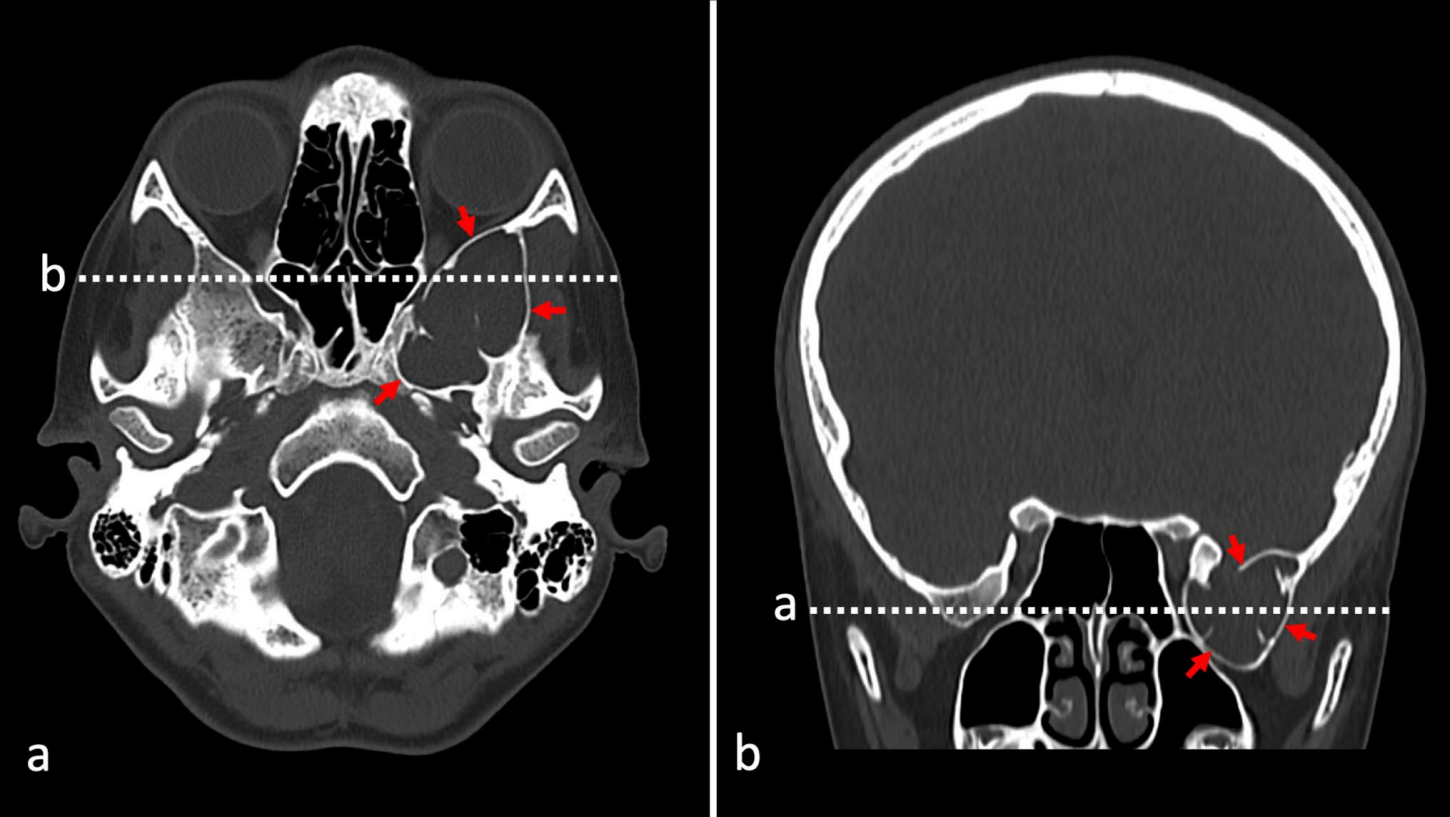
Preoperative non-enhanced head CT scan axial cross-section (**a**) with white dotted line marking level of the coronal section (**b**). Cyst is marked with red arrows

A 12-year-old male patient has been admitted to the emergency department due to a mild head injury without loss of consciousness. He has been complaining of headaches in the parietooccipital area caused by direct trauma. He did not present nausea, double vision, or any other sign or symptom. There was no medical history of previous traumatic accidents, chronic diseases, allergies, and undergone procedures, including dental. During a neurological examination, he has presented no focal deficits. Non-contrast CT has revealed no acute post-traumatic intracranial findings, such as bone fracture (neither base of the skull nor calvaria), intracranial bleeding, signs of cerebral edema, and midline shift. In the area of the floor of the middle cranial fossa on the left side, an accidental finding was made. Large, (approximate dimensions, 3.3 cm ap × 2.6 cm cor × 2.5 cm cc) hypodense lesion of cerebrospinal fluid (CSF) attenuation was visualized within a greater wing of sphenoid bone (Fig. 1).

**Fig. 2:**
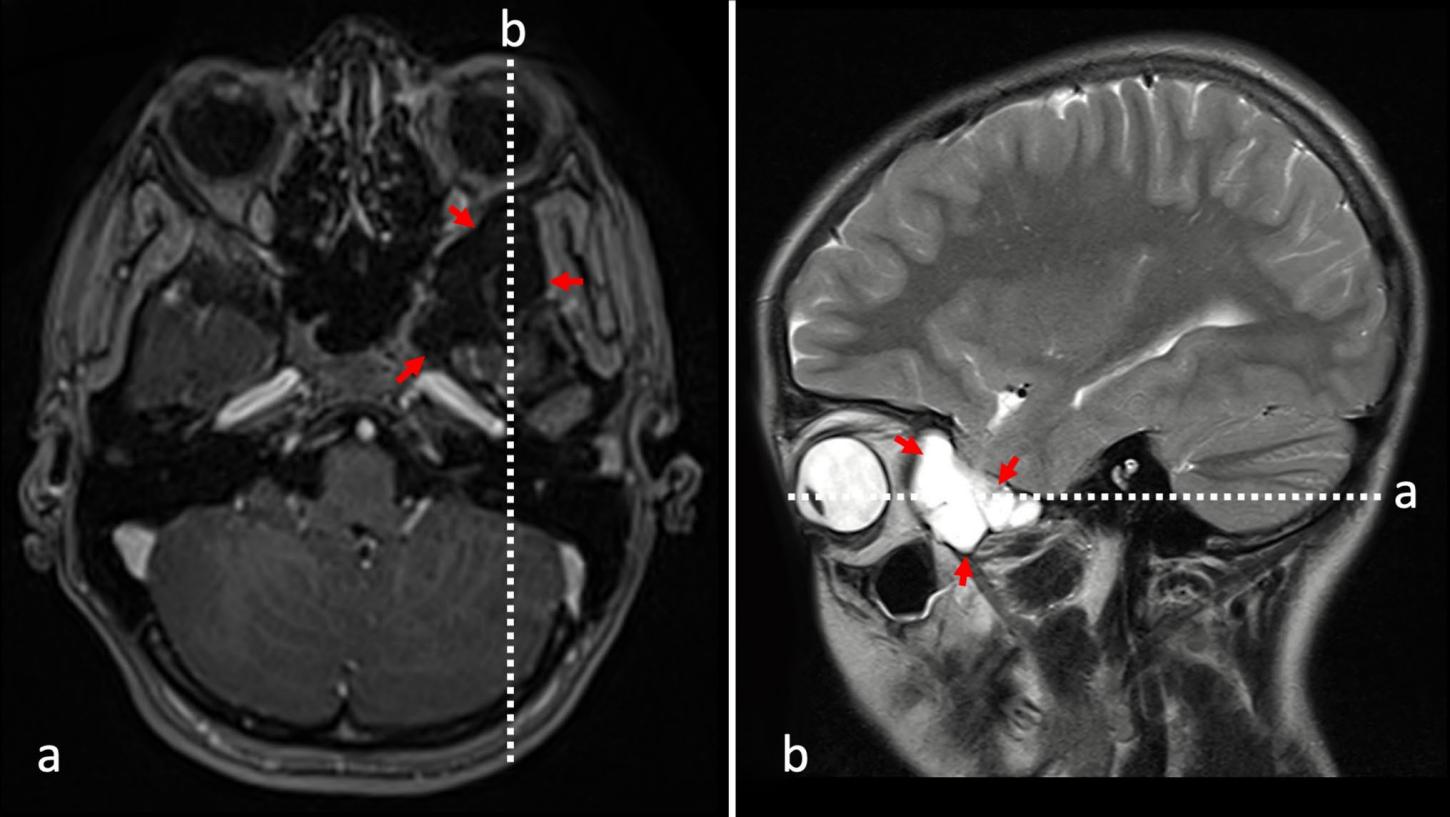
Preoperative head MRI T1W contrast-enhanced scan on axial cross-section (**a**) with white dotted line marking level of the sagittal cross-section (**b**) of the T2W image. Cyst is marked with red arrows

The lesion was limited with generally dense, cortical bone wall, occasionally thin and spiculated. There was no component of associated thickening of soft tissue. The osseous wall of surrounding cranial spaces such as the sphenoid and maxillary sinus, orbit, and nasal cavity appeared intact. There was no radiologic evidence for the destruction of cranial base foramina and fissures (i.e., foramen rotundum, foramen ovale, foramen spinosum, inferior orbital fissure). MRI revealed irregular space with CSF intensity signal: low in T1W, FLAIR, high in T2W (Fig. 2).

No restriction of diffusion was described in diffusion-weighted (DW) sequence. In addition, the connection between the inside of the lesion and subarachnoid space was discovered and visualized in the region of the slight absence of the osseous floor of the middle cranial fossa. Based on the clinical status and radiological findings, the patient was qualified for surgical intervention.

## Management

The pterional approach was performed—the periosteum and temporalis muscle have been cut in a typical fashion and the periosteal flap was preserved for further reconstruction. Rectangle-shaped craniotomy has been made, and the dura of the middle cranial fossa has been shown.

**Fig. 3:**
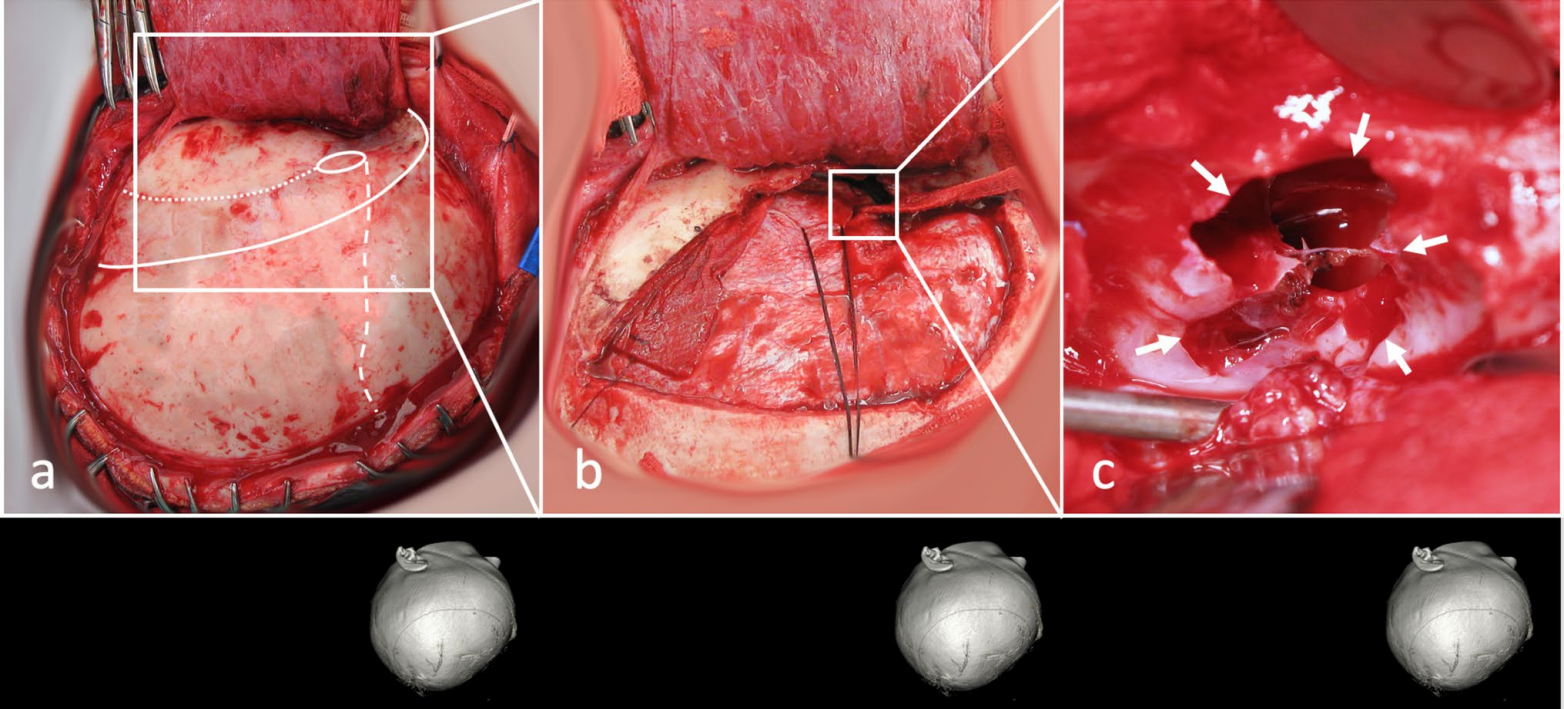
Intraoperative view. Characteristic craniometrical points and lines such as pterion (circle), coronal suture (intermittent line), squamous suture (dotted line), and superior temporal line (continuous line) were marked on the skull (**a**). Pterional craniotomy was performed, and periosteal flap was preserved (**b**). After dissection and mobilization of the dura, intradiploic space was visualized. Cyst is marked with white arrows (**c**)

The dissection has been continued medially until visualization of the communicating channel between the dura on the floor of the cranial fossa and the intradiploic space of the greater wing of the sphenoid bone (Fig. 3).

The superior osseous wall of the cyst has been drilled, and the arachnoid-like tissue covering the cyst inside has been removed and preserved for histopathology examination as well as the fluid content of the cyst. Subdural space has been revised and duroplasty has been performed with the use of artificial materials. Intradiploic space has been filled with periosteal flap.

The were no abnormal findings described in the post-op CT scan. During the post-operative period, no complications such as CSF leakage, wound infection, or excessive pain complaints were observed. The patient was dismissed in good general condition, with no neurological deficits.

Histopathology examination of the specimen revealed that the cyst wall consisted of the membrane of leptomeninx and dura mater cells. Based on radiological, surgical, and histopathological findings, the diagnosis of intradiploic arachnoid cyst has been made.

## Discussion

Bright was the first who defined, in 1831, a group of specific intracranial findings as “serous cysts forming in connection with the arachnoid and apparently lying between its layers” based on cadaveric studies [5]. As modern radiologic tools have become more available, ACs have been widely described in the literature. Well-developed prenatal diagnostics and increased availability of CT/MR imaging are the cause of early detection of arachnoid cysts—most of them are recognized before an individual reaches 30 years of age [5]. ACs are most commonly discovered incidentally while running diagnostics of pathologies unrelated to the location and development of the arachnoid cyst and post-traumatic events [6, 7].

The characteristic symptoms associated with ACs were not revealed in a population-based study [8]; however, the most common include headache, weakness, seizures, and hydrocephalus [5]. On the other hand, headache is one of the most common complaints in the population; thus, its coexistence with arachnoid cyst may be simply accidental. This approach may also be supported by cases in which spontaneous improvement has been observed without any treatment [9]. In a group of pediatric patients with confirmed presence of ACs, the most common primary indications for radiological diagnostics were seizure, headache, developmental delay, and acute mental status change [10].

One of the most important factors that correlates with the symptomatic appearance of ACs is the localization of the lesion. A higher risk of symptomatic presentation has suprasellar region, cerebello-pontine angle, ambient cistern, or quadrigeminal cistern [9]. The infratentorial location has frequent association with other intra- or extracranial developmental abnormalities as investigated by Harrison [11], McCullough et al. [12], or Menezes et al. [13].

There are many classification systems of arachnoid cysts and currently the most widely used was proposed by Galassi et al. [14]. This system divides ACs into 3 types, with type I as the mildest and type III as the most severe [14]. One must bear in mind Galassi’s classification describes arachnoid cysts located in the middle cranial fossa only.

Considering the etiopathogenesis of these phenomena, ACs can be divided into two large groups:

1. Primary—occur more often than secondary; these are caused by congenital malformation developing during gestation such as duplication or splitting of the arachnoid mater; further expansion of the cyst can occur because of 3 possible mechanisms [15]:
  Fluid production by the cyst wall,
  A ball-valve mechanism,
  Osmotic gradient between the cyst and subarachnoid space.
2. Secondary—developing as an effect of potentially harmful or infectious factors (i.e., meningitis, head injury, tumor, surgical intervention) [7].

Despite common histological features, the intraosseous or intradiploic arachnoid cyst should be considered a separate clinical issue. The key factor for such an entity to arise is the discontinuity of the inner cortical bone of the skull. It may involve any bone of the calvaria or the base of the skull [6]. Another essential feature is a rupture of the dura—it facilitates breaching out of the arachnoid membrane and expanding into the diploe [4]. It seems obligatory since we did not find any description in the literature of the intradiploic and simultaneously intradural cyst. Primary intraosseous expansion of the arachnoid matter is probably an effect of CSF entering the diploe through the preexisting dehiscences or natural foramina with the force of intracranial pressure [6]. However, the non-traumatic variety of intradiploic cyst is very rare—usually seen in the occipital region, the temporal and frontal skull base [16, 17].

The secondary ACs develop usually at the base of the trauma that causes bone fracture and tears the dura. When both the inner and outer table of the skull is fractured, invagination of the arachnoid mater into the osseous defect creates an obstacle on the way of healing the fracture. If that happens, the complication of head injury known as growing skull fracture may occur. It is usually seen in childhood and contributes to an incidence of 0.05–1.6% of pediatric skull fractures [18] (Table 1).

**Table 1:** Etiopathogenetic division of intradiploic arachnoid cysts or arachnoid diverticulae according to our terminology

Primary	Secondary
Arachnoid matter entering the diploe through the preexisting dehiscences or natural foramina with the force of intracranial pressure	Tearing the dura by the skull fracture and invagination of the arachnoid into the osseous defect

We assume this is the first description of large intrasphenoid AC without any traumatic or iatrogenic cause. The literature provides many different terms for the phenomenon. D’Almeida and King [19] named that *intradiploic cerebrospinal fluid fistula*, whereas Weinand et al. [4] were the first who used the term *intradiploic arachnoid cyst* in 1989. Among others, there are *leptomeningeal cyst* or *post-traumatic arachnoid cyst* [1]. As the intradiploic arachnoid membrane can be compared to a pouch opening from one space to another, we are proposing the term *intradiploic arachnoid diverticulum* as the more accurate for capturing the essence of the phenomenon. It provides clear differentiation of the entities from classical arachnoid cysts since they are of different anatomical localization (intradural vs. extradural) and etiopathogenesis. Our comparison of all those findings is presented in Table 2.

**Table 2:** Comparison of arachnoid cysts and arachnoid diverticulae

	**Arachnoid cysts**	**Intradiploic arachnoid diverticulae**
Localization	Intradural	Extradural
Etiopathogenesis	Usually primary—caused by congenital malformation developing during gestation	Usually secondary—develops at the base of the trauma that causes bone fracture and tears the dura
Treatment	Not required usually	Can be required when complicated with growing skull fracture

CT scans followed by MRI give quite obvious radiologic visualization of the arachnoid cysts and diverticula. However, during the management of AC-like lesions, one should consider dermoid or epidermoid cyst, mucocele, aneurysmal bone cyst, plasmacytoma, metastasis, or cystic fibrous dysplasia [1, 4]. Sharma et al. [20] in their report of a giant intradiploic cyst of occipital bone advised exploratory surgery, which was not performed because of patient’s refusal. In our case, the decision of surgical treatment was made based on the radiological appearance. The uncommon localization of the feature and bone destruction around it led the authors to decide about the surgery. The lack of appropriate recommendations and descriptions of the management of such entities in the literature is unsatisfactory. Our present experience gives us a new perspective on the treatment of asymptomatic arachnoid cysts, and we would like to recommend observation with serial imaging studies, for example, with non-enhanced MRI after 6–12 months.

## Conclusions

- ACs may be asymptomatic and usually they are discovered incidentally while running diagnostics after head trauma.
- Non-contrast CT scans followed by basic sequences of MR imaging are often enough in terms of differential diagnosis.
- Surgical treatment may be necessary in case of symptomatic ACs.
- We propose a new term—*intradiploic arachnoid diverticulum*—for intraosseous localization of arachnoid entities.
- Treatment of arachnoid diverticulum is not yet established, but observation with serial imaging studies should be recommended as primary management in case of asymptomatic cyst.

## Data Availability

Please contact authors for data requests (Adrian Drożdż MD—email: adrian.drozdz@wum.edu.pl). The data belong to the Department of Pediatric Neurosurgery, Bogdanowicz Memorial Hospital for Children, and are not available to share unless in the form included in the manuscript.
